# (2*Z*)-3-(2,4-Di­chloro­phen­yl)-3-hy­droxy-*N*-phenyl­prop-2-ene­thio­amide

**DOI:** 10.1107/S1600536813017339

**Published:** 2013-06-29

**Authors:** Dalbir Kour, Kuldeep Singh, Mayur M. Aitawade, Madhukar B. Deshmukh, Prashant V. Anbhule, Vivek K. Gupta, Rajni Kant

**Affiliations:** a X-ray Crystallography Laboratory, Post-Graduate Department of Physics & Electronics, University of Jammu, Jammu Tawi 180 006, India; b Department of Agrochemicals & Pest Management, Shivaji University, Kolhapur, India; c Department of Chemistry, Shivaji University, Kolhapur, India

## Abstract

In the title mol­ecule, C_15_H_11_Cl_2_NOS, the dihedral angle between the phenyl and benzene rings is 72.24 (1)°. In the crystal, pairs of N—H⋯S hydrogen bonds form dimers with twofold rotational symmetry. The dimers are connected by weak C—H⋯O hydrogen bonds, forming a two-dimensional network parallel to (001). An intra­molecular O—H⋯S hydrogen bond is also observed.

## Related literature

For the biological activity and applications of thio­amides, see: Zahid *et al.* (2009 ▶); Jagodzinski (2003 ▶); Lebana *et al.* (2008 ▶). For the synthesis of thio­amides, see: Bauer & Kuhlein (1985 ▶); Cava & Levinson (1985 ▶). For the synthesis of the title compound, see: Rudrof *et al.* (1979 ▶). For related structures, see: Xu *et al.* (2005 ▶); Cowley *et al.* (2002 ▶); Jiang (2009 ▶); Patil *et al.* (2011 ▶); Deshmukh *et al.* (2009 ▶). For standard bond-length data, see: Allen *et al.* (1987 ▶).
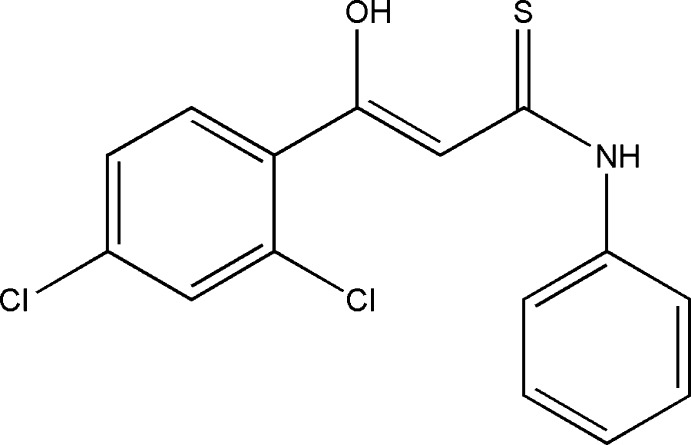


## Experimental

### 

#### Crystal data

C_15_H_11_Cl_2_NOS
*M* *_r_* = 324.21
Orthorhombic, 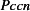

*a* = 28.9562 (6) Å
*b* = 13.2610 (3) Å
*c* = 7.5284 (2) Å
*V* = 2890.82 (12) Å^3^
*Z* = 8
Mo *K*α radiation
μ = 0.59 mm^−1^
*T* = 293 K
0.3 × 0.2 × 0.1 mm

#### Data collection

Agilent Xcalibur Sapphire3 diffractometer
Absorption correction: multi-scan (*CrysAlis PRO*; Oxford Diffraction, 2010 ▶) *T*_min_ = 0.835, *T*_max_ = 1.000
63107 measured reflections
2836 independent reflections
2275 reflections with *I* > 2σ(*I*)
*R*_int_ = 0.067

#### Refinement

*R*[*F* ^2^ > 2σ(*F* ^2^)] = 0.044
*wR*(*F* ^2^) = 0.091
*S* = 1.11
2836 reflections
185 parameters
H atoms treated by a mixture of independent and constrained refinement
Δρ_max_ = 0.22 e Å^−3^
Δρ_min_ = −0.18 e Å^−3^

### 

Data collection: *CrysAlis PRO* (Oxford Diffraction, 2010 ▶); cell refinement: *CrysAlis PRO*; data reduction: *CrysAlis PRO*; program(s) used to solve structure: *SHELXS97* (Sheldrick, 2008 ▶); program(s) used to refine structure: *SHELXL97* (Sheldrick, 2008 ▶); molecular graphics: *ORTEP-3 for Windows* (Farrugia, 2012 ▶) and *Mercury* (Macrae *et al.*, 2008 ▶); software used to prepare material for publication: *PLATON* (Spek, 2009 ▶).

## Supplementary Material

Crystal structure: contains datablock(s) I, global. DOI: 10.1107/S1600536813017339/lh5625sup1.cif

Structure factors: contains datablock(s) I. DOI: 10.1107/S1600536813017339/lh5625Isup2.hkl

Supplementary material file. DOI: 10.1107/S1600536813017339/lh5625Isup3.cml

Additional supplementary materials:  crystallographic information; 3D view; checkCIF report

## Figures and Tables

**Table 1:** Hydrogen-bond geometry (Å, °)

*D*—H⋯*A*	*D*—H	H⋯*A*	*D*⋯*A*	*D*—H⋯*A*
N1—H1⋯S1^i^	0.86	2.61	3.4397 (18)	162
C3′—H3′⋯O1^ii^	0.93	2.59	3.496 (3)	164
O1—H11⋯S1	0.89 (3)	2.10 (3)	2.9315 (18)	157 (2)

Symmetry codes: (i) 
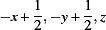
; (ii) 
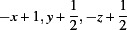
.

**Fig. 1.**
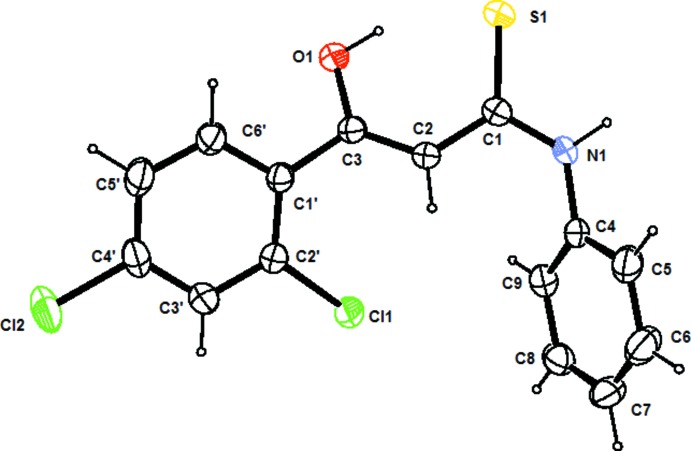
The molecular structure of (I) with displacement ellipsoids drawn at the 40% probability level. H atoms are shown as small spheres of arbitrary radii.

**Fig. 2.**
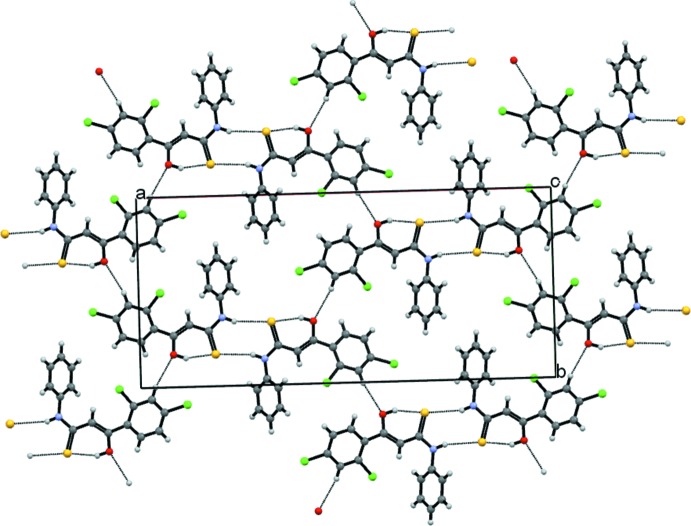
Part of the crystal structure with hydrogen bonds shown as dotted lines.

## supplementary crystallographic information

### Comment

Thioamides exhibit a wide range of applications, not only as synthetic intermediates in the synthesis of a variety of hetero-cyclic compounds (Zahid *et al.*, 2009), but also numerous biological activities have been associated with them (Jagodzinski, 2003). Moreover, thioamides are important ligands in the field of coordination chemistry (Lebana *et al.*, 2008). Several synthetic reports on thioamides have been published involving the uses of Lawesson's regent (Cava & Levinson, 1985) and phosphorus pentasulfide (Bauer & Kuhlein, 1985). Our ongoing research involves the development of newer synthetic methodologies for heterocyclic compounds (Patil *et al.*, 2011; Deshmukh *et al.*, 2009). The crystal structure of the title compound is described herein.

The molecular structure of the title compound (I) is shown in Fig. 1. The bond lengths (Allen, *et al.*, 1987) and angles observed in (I) show normal values and are comparable with related structures (Xu, *et al.*, 2005; Jiang, 2009). The dihedral angle between the phenyl and benzene rings [C1'-C6' and C4-C9] is 72.24 (1)°. The two chlorine atoms Cl1 and Cl2 which were not included in the calculation of the least-squares plane of the C1'-C6' ring, deviate from the plane by 0.1336 (1) and 0.0310 (1) Å. The C1—S1 bond length of 1.695 (2) Å is comparable with the value [1.688 (2) Å] in a related structure (Cowley *et al.*, 2002). In the crystal, pairs of N—H···S hydrogen bonds form dimers with twofold rotational symmetry. The dimers are connected by weak C—H···O hydrogen bonds to form a two-dimensional network parallel to (001). An intramolecular O—H···S hydrogen bond is also observed. The hydrogen bonds are shown in Fig. 2.

### Experimental

(2*Z*)-3-(2,4-dichlorophenyl)-3-hydroxy-*N*-phenylprop-2-enethioamide was synthesized by a previously reported procedure (Rudrof *et al.*, 1979). The product dissolved in EtOH, on slow evaporation of the solvent formed crystals of the title compound. Yield: 85%. IR (KBr): 3442, 3207, 1607, 1364, 1224 cm-1. 1H NMR (300 MHz, CDCl3): dH = 5.97 (s, 1H, CH), 7.19–7.53 (m, 8H, Ar—H),8.29 (bs, 1H, NH), 14.75 (s, 1H, OH). 13 C NMR (CDCl3): dc = 136.3, 133.7, 132.6, 131.2, 137.0, 130.9, 129.4, 128.8, 127.5, 127.1, 126.8, 125.2, 123.1. (m/*z*) = 324.

### Refinement

Hydrogen atom H11 bonded to O1 was located in a difference Fourier map and was refined independently with an isotropic displacement parameter. All other H atoms were positioned geometrically and were treated as riding on their parent atoms, with C—H = 0.93 Å, N—H = 0.86Å and *U*_iso_(H)=1.2*U*_eq_(C,N).

### Crystal data

**Table:**

C_15_H_11_Cl_2_NOS	*F*(000) = 1328
*M**_r_* = 324.21	*D*_x_ = 1.490 Mg m^−^^3^
Orthorhombic, *P**c**c**n*	Mo *K*α radiation, λ = 0.71073 Å
Hall symbol: -P 2ab 2ac	Cell parameters from 24816 reflections
*a* = 28.9562 (6) Å	θ = 3.4–29.1°
*b* = 13.2610 (3) Å	µ = 0.59 mm^−^^1^
*c* = 7.5284 (2) Å	*T* = 293 K
*V* = 2890.82 (12) Å^3^	Block, orange
*Z* = 8	0.3 × 0.2 × 0.1 mm

### Data collection

**Table:**

Agilent Xcalibur Sapphire3 diffractometer	2836 independent reflections
Radiation source: fine-focus sealed tube	2275 reflections with *I* > 2σ(*I*)
Graphite monochromator	*R*_int_ = 0.067
Detector resolution: 16.1049 pixels mm^-1^	θ_max_ = 26.0°, θ_min_ = 3.4°
ω scan	*h* = −35→35
Absorption correction: multi-scan (*CrysAlis PRO*; Oxford Diffraction, 2010)	*k* = −16→16
*T*_min_ = 0.835, *T*_max_ = 1.000	*l* = −9→9
63107 measured reflections	

### Refinement

**Table:**

Refinement on *F*^2^	Primary atom site location: structure-invariant direct methods
Least-squares matrix: full	Secondary atom site location: difference Fourier map
*R*[*F*^2^ > 2σ(*F*^2^)] = 0.044	Hydrogen site location: inferred from neighbouring sites
*wR*(*F*^2^) = 0.091	H atoms treated by a mixture of independent and constrained refinement
*S* = 1.11	*w* = 1/[σ^2^(*F*_o_^2^) + (0.0293*P*)^2^ + 2.0935*P*] where *P* = (*F*_o_^2^ + 2*F*_c_^2^)/3
2836 reflections	(Δ/σ)_max_ = 0.001
185 parameters	Δρ_max_ = 0.22 e Å^−^^3^
0 restraints	Δρ_min_ = −0.18 e Å^−^^3^

### Special details

**Table:**

Experimental. *CrysAlis PRO*, Oxford Diffraction Ltd., Version 1.171.34.40 (release 27–08-2010 CrysAlis171. NET) (compiled Aug 27 2010,11:50:40) Empirical absorption correction using spherical harmonics, implemented in SCALE3 ABSPACK scaling algorithm.
Geometry. All e.s.d.'s (except the e.s.d. in the dihedral angle between two l.s. planes) are estimated using the full covariance matrix. The cell e.s.d.'s are taken into account individually in the estimation of e.s.d.'s in distances, angles and torsion angles; correlations between e.s.d.'s in cell parameters are only used when they are defined by crystal symmetry. An approximate (isotropic) treatment of cell e.s.d.'s is used for estimating e.s.d.'s involving l.s. planes.
Refinement. Refinement of *F*^2^ against ALL reflections. The weighted *R*-factor *wR* and goodness of fit *S* are based on *F*^2^, conventional *R*-factors *R* are based on *F*, with *F* set to zero for negative *F*^2^. The threshold expression of *F*^2^ > σ(*F*^2^) is used only for calculating *R*-factors(gt) *etc*. and is not relevant to the choice of reflections for refinement. *R*-factors based on *F*^2^ are statistically about twice as large as those based on *F*, and *R*- factors based on ALL data will be even larger.

### Fractional atomic coordinates and isotropic or equivalent isotropic displacement parameters (Å^2^)

**Table:**

	*x*	*y*	*z*	*U*_iso_*/*U*_eq_	
S1	0.31822 (2)	0.16889 (5)	0.20313 (10)	0.04414 (18)	
O1	0.41937 (6)	0.17165 (12)	0.2188 (3)	0.0476 (5)	
N1	0.29969 (6)	0.36160 (14)	0.2171 (3)	0.0391 (5)	
H1	0.2719	0.3391	0.2114	0.047*	
Cl1	0.44813 (2)	0.49703 (5)	0.15039 (11)	0.0581 (2)	
Cl2	0.61091 (2)	0.40683 (7)	0.43216 (13)	0.0718 (3)	
C1	0.33303 (8)	0.29196 (17)	0.2249 (3)	0.0338 (5)	
C1'	0.46507 (7)	0.31015 (16)	0.2953 (3)	0.0320 (5)	
C2	0.37934 (7)	0.32640 (17)	0.2576 (3)	0.0333 (5)	
H2	0.3828	0.3945	0.2840	0.040*	
C2'	0.48099 (8)	0.40787 (17)	0.2622 (3)	0.0360 (5)	
C3	0.41830 (7)	0.27056 (16)	0.2540 (3)	0.0315 (5)	
C3'	0.52538 (8)	0.43770 (19)	0.3058 (3)	0.0430 (6)	
H3'	0.5351	0.5033	0.2834	0.052*	
C4	0.30453 (7)	0.46842 (17)	0.2172 (3)	0.0343 (5)	
C4'	0.55494 (8)	0.3693 (2)	0.3827 (3)	0.0440 (6)	
C5	0.28010 (8)	0.5236 (2)	0.3398 (4)	0.0463 (6)	
H5	0.2619	0.4912	0.4241	0.056*	
C5'	0.54086 (8)	0.2721 (2)	0.4168 (4)	0.0478 (7)	
H5'	0.5610	0.2261	0.4685	0.057*	
C6	0.28268 (10)	0.6273 (2)	0.3371 (4)	0.0565 (8)	
H6	0.2661	0.6647	0.4200	0.068*	
C6'	0.49660 (8)	0.2442 (2)	0.3733 (3)	0.0404 (6)	
H6'	0.4873	0.1784	0.3969	0.048*	
C7	0.30932 (10)	0.6760 (2)	0.2137 (4)	0.0534 (7)	
H7	0.3111	0.7460	0.2134	0.064*	
C8	0.33346 (9)	0.6208 (2)	0.0902 (4)	0.0476 (7)	
H8	0.3515	0.6537	0.0062	0.057*	
C9	0.33111 (8)	0.51666 (18)	0.0900 (3)	0.0401 (6)	
H9	0.3472	0.4795	0.0055	0.048*	
H11	0.3902 (11)	0.152 (2)	0.210 (4)	0.070 (10)*	

### Atomic displacement parameters (Å^2^)

**Table:**

	*U*^11^	*U*^22^	*U*^33^	*U*^12^	*U*^13^	*U*^23^
S1	0.0340 (3)	0.0307 (3)	0.0677 (4)	−0.0066 (3)	0.0007 (3)	0.0007 (3)
O1	0.0327 (10)	0.0286 (9)	0.0816 (13)	0.0008 (7)	0.0009 (9)	−0.0031 (9)
N1	0.0216 (10)	0.0322 (10)	0.0636 (14)	−0.0034 (8)	−0.0027 (9)	0.0025 (10)
Cl1	0.0339 (3)	0.0381 (3)	0.1022 (6)	−0.0011 (3)	−0.0061 (4)	0.0241 (4)
Cl2	0.0333 (4)	0.0834 (6)	0.0989 (6)	−0.0046 (4)	−0.0186 (4)	−0.0110 (5)
C1	0.0319 (13)	0.0313 (12)	0.0382 (13)	−0.0035 (10)	0.0017 (10)	0.0014 (10)
C1'	0.0279 (11)	0.0327 (12)	0.0354 (12)	0.0031 (9)	0.0028 (9)	0.0003 (10)
C2	0.0292 (12)	0.0258 (11)	0.0451 (13)	−0.0016 (9)	0.0008 (10)	0.0000 (10)
C2'	0.0298 (12)	0.0331 (12)	0.0452 (13)	0.0025 (10)	0.0006 (10)	0.0015 (11)
C3	0.0303 (12)	0.0268 (12)	0.0375 (12)	0.0001 (9)	0.0028 (10)	0.0023 (10)
C3'	0.0322 (13)	0.0378 (13)	0.0591 (16)	−0.0023 (11)	−0.0001 (12)	−0.0034 (12)
C4	0.0236 (11)	0.0310 (12)	0.0484 (14)	0.0020 (9)	−0.0073 (10)	0.0016 (11)
C4'	0.0270 (13)	0.0569 (16)	0.0480 (15)	−0.0003 (11)	−0.0039 (11)	−0.0075 (13)
C5	0.0357 (14)	0.0479 (15)	0.0552 (16)	0.0073 (11)	0.0058 (12)	0.0036 (13)
C5'	0.0363 (14)	0.0518 (17)	0.0552 (16)	0.0091 (12)	−0.0079 (12)	0.0076 (13)
C6	0.0555 (18)	0.0457 (16)	0.069 (2)	0.0163 (14)	−0.0018 (15)	−0.0087 (15)
C6'	0.0343 (13)	0.0391 (13)	0.0477 (14)	0.0028 (11)	0.0003 (11)	0.0094 (12)
C7	0.0519 (17)	0.0324 (14)	0.076 (2)	0.0054 (12)	−0.0157 (15)	0.0011 (14)
C8	0.0381 (14)	0.0434 (15)	0.0613 (17)	−0.0052 (11)	−0.0074 (13)	0.0148 (13)
C9	0.0301 (13)	0.0420 (14)	0.0481 (14)	0.0000 (10)	−0.0002 (11)	0.0021 (12)

### Geometric parameters (Å, º)

**Table:**

S1—C1	1.695 (2)	C3'—H3'	0.9300
O1—C3	1.339 (3)	C4—C5	1.374 (3)
O1—H11	0.89 (3)	C4—C9	1.385 (3)
N1—C1	1.337 (3)	C4'—C5'	1.376 (4)
N1—C4	1.423 (3)	C5—C6	1.377 (4)
N1—H1	0.8600	C5—H5	0.9300
Cl1—C2'	1.735 (2)	C5'—C6'	1.374 (3)
Cl2—C4'	1.736 (2)	C5'—H5'	0.9300
C1—C2	1.438 (3)	C6—C7	1.369 (4)
C1'—C6'	1.394 (3)	C6—H6	0.9300
C1'—C2'	1.398 (3)	C6'—H6'	0.9300
C1'—C3	1.485 (3)	C7—C8	1.374 (4)
C2—C3	1.350 (3)	C7—H7	0.9300
C2—H2	0.9300	C8—C9	1.382 (3)
C2'—C3'	1.384 (3)	C8—H8	0.9300
C3'—C4'	1.375 (3)	C9—H9	0.9300
			
C3—O1—H11	106 (2)	C3'—C4'—C5'	120.8 (2)
C1—N1—C4	128.05 (19)	C3'—C4'—Cl2	118.8 (2)
C1—N1—H1	116.0	C5'—C4'—Cl2	120.3 (2)
C4—N1—H1	116.0	C4—C5—C6	119.6 (3)
N1—C1—C2	117.5 (2)	C4—C5—H5	120.2
N1—C1—S1	118.57 (17)	C6—C5—H5	120.2
C2—C1—S1	123.93 (17)	C6'—C5'—C4'	119.0 (2)
C6'—C1'—C2'	116.2 (2)	C6'—C5'—H5'	120.5
C6'—C1'—C3	117.6 (2)	C4'—C5'—H5'	120.5
C2'—C1'—C3	126.2 (2)	C7—C6—C5	120.7 (3)
C3—C2—C1	127.0 (2)	C7—C6—H6	119.6
C3—C2—H2	116.5	C5—C6—H6	119.6
C1—C2—H2	116.5	C5'—C6'—C1'	122.8 (2)
C3'—C2'—C1'	121.9 (2)	C5'—C6'—H6'	118.6
C3'—C2'—Cl1	115.43 (18)	C1'—C6'—H6'	118.6
C1'—C2'—Cl1	122.49 (18)	C6—C7—C8	119.7 (3)
O1—C3—C2	124.1 (2)	C6—C7—H7	120.2
O1—C3—C1'	111.51 (18)	C8—C7—H7	120.2
C2—C3—C1'	124.3 (2)	C7—C8—C9	120.5 (3)
C4'—C3'—C2'	119.3 (2)	C7—C8—H8	119.7
C4'—C3'—H3'	120.3	C9—C8—H8	119.7
C2'—C3'—H3'	120.3	C8—C9—C4	119.2 (2)
C5—C4—C9	120.3 (2)	C8—C9—H9	120.4
C5—C4—N1	118.7 (2)	C4—C9—H9	120.4
C9—C4—N1	120.9 (2)		
			
C4—N1—C1—C2	7.8 (4)	C1—N1—C4—C9	56.9 (4)
C4—N1—C1—S1	−174.3 (2)	C2'—C3'—C4'—C5'	−0.1 (4)
N1—C1—C2—C3	−173.3 (2)	C2'—C3'—C4'—Cl2	178.55 (19)
S1—C1—C2—C3	8.9 (4)	C9—C4—C5—C6	−0.9 (4)
C6'—C1'—C2'—C3'	−0.6 (3)	N1—C4—C5—C6	−177.1 (2)
C3—C1'—C2'—C3'	180.0 (2)	C3'—C4'—C5'—C6'	−0.2 (4)
C6'—C1'—C2'—Cl1	174.85 (18)	Cl2—C4'—C5'—C6'	−178.9 (2)
C3—C1'—C2'—Cl1	−4.6 (3)	C4—C5—C6—C7	0.0 (4)
C1—C2—C3—O1	−0.2 (4)	C4'—C5'—C6'—C1'	0.1 (4)
C1—C2—C3—C1'	−177.8 (2)	C2'—C1'—C6'—C5'	0.3 (4)
C6'—C1'—C3—O1	−29.6 (3)	C3—C1'—C6'—C5'	179.7 (2)
C2'—C1'—C3—O1	149.8 (2)	C5—C6—C7—C8	0.5 (4)
C6'—C1'—C3—C2	148.2 (2)	C6—C7—C8—C9	−0.1 (4)
C2'—C1'—C3—C2	−32.4 (4)	C7—C8—C9—C4	−0.8 (4)
C1'—C2'—C3'—C4'	0.5 (4)	C5—C4—C9—C8	1.3 (4)
Cl1—C2'—C3'—C4'	−175.2 (2)	N1—C4—C9—C8	177.4 (2)
C1—N1—C4—C5	−126.9 (3)		

### Hydrogen-bond geometry (Å, º)

**Table:**

*D*—H···*A*	*D*—H	H···*A*	*D*···*A*	*D*—H···*A*
N1—H1···S1^i^	0.86	2.61	3.4397 (18)	162
C3′—H3′···O1^ii^	0.93	2.59	3.496 (3)	164
O1—H11···S1	0.89 (3)	2.10 (3)	2.9315 (18)	157 (2)

Symmetry codes: (i) −*x*+1/2, −*y*+1/2, *z*; (ii) −*x*+1, *y*+1/2, −*z*+1/2.

## References

[bb1] Allen, F. H., Kennard, O., Watson, D. G., Brammer, L., Orpen, A. G. & Taylor, R. (1987). *J. Chem. Soc. Perkin Trans. 2*, pp. S1–19.

[bb2] Bauer, W. & Kuhlein, K. (1985). *Houben–Weyl Methoden der Organischen Chemie*, Vol. E5, p. 1218. Stuttgart, New York: Georg Thieme Verlag.

[bb3] Cava, M. P. & Levinson, M. I. (1985). *Tetrahedron*, **41**, 5061–5087.

[bb4] Cowley, A. R., Dilworth, J. R. & Dorinelly, P. S. (2002). *J. Am. Chem. Soc.* **124**, 5270–5271. 10.1021/ja012668z 11996559

[bb5] Deshmukh, M. B., Salunkhe, S. M., Patil, D. R. & Anbhule, P. V. (2009). *Eur. J. Med. Chem.* **44**, 2651–2654. 10.1016/j.ejmech.2008.10.018 19036478

[bb6] Farrugia, L. J. (2012). *J. Appl. Cryst.* **45**, 849–854.

[bb7] Jagodzinski, T. S. (2003). *Chem. Rev.* **103**, 197–227. 10.1021/cr0200015 12517184

[bb8] Jiang, J.-H. (2009). *Acta Cryst.* E**65**, o52. 10.1107/S1600536808040907 PMC2967966 21581693

[bb9] Lebana, S. T., Sultana, R. & Hendal, G. (2008). *Polyhedron*, **27**, 1008–1016.

[bb10] Macrae, C. F., Bruno, I. J., Chisholm, J. A., Edgington, P. R., McCabe, P., Pidcock, E., Rodriguez-Monge, L., Taylor, R., van de Streek, J. & Wood, P. A. (2008). *J. Appl. Cryst.* **41**, 466–470.

[bb11] Oxford Diffraction (2010). *CrysAlis PRO* Oxford Diffraction Ltd, Yarnton, England.

[bb12] Patil, D. R., Salunkhe, S. M., Aitawade, M. M., Deshmukh, M. B., Kolekar, G. B. & Anbhule, P. V. (2011). *Pharma Chem.* **3**, 207–214.

[bb13] Rudrof, W. D., Schierhorn, A. & Augustin, M. (1979). *Tetrahedron*, **35**, 551–556.

[bb14] Sheldrick, G. M. (2008). *Acta Cryst.* A**64**, 112–122. 10.1107/S0108767307043930 18156677

[bb15] Spek, A. L. (2009). *Acta Cryst.* D**65**, 148–155. 10.1107/S090744490804362X PMC2631630 19171970

[bb16] Xu, L.-Z., Yang, S.-H., Zhu, C.-Y., Li, K. & Liu, F.-Q. (2005). *Acta Cryst.* E**61**, o259–o260.

[bb17] Zahid, M., Yasin, K. A., Akhtar, T., Rama, N. H., Hameed, S., Al Masoudi, N. A., Loddo, R. & La Colla, P. (2009). *Arkivoc*, **xi**, 85–93.

